# Patient‐derived ovarian cancer organoid carries immune microenvironment and blood vessel keeping high response to cisplatin

**DOI:** 10.1002/mco2.697

**Published:** 2024-08-28

**Authors:** Yuqing Zhao, Chen Wang, Wei Deng, Lanyang Li, Jiping Liu, Yanghua Shi, Xiang Tao, Jian Zhang, Qi Cao, Chunhui Cai, Xinxin Han

**Affiliations:** ^1^ Obstetrics & Gynecology Hospital Fudan University Shanghai China; ^2^ Department of Research Shanghai LiSheng Biotech Shanghai China; ^3^ LongHua Hospital Shanghai University of Traditional Chinese Medicine Shanghai China; ^4^ Department of Oncology Shanghai Ninth People's Hospital Shanghai Jiao Tong University School of Medicine Shanghai China; ^5^ Organ Regeneration X Lab LiSheng East China Institute of Biotechnology Peking University Jiangsu China

**Keywords:** cisplatin, organoid, ovarian cancer, tumor microenvironment (TME), vascularization

## Abstract

Ovarian cancer is high recurrence and mortality malignant tumor. The most common ovarian cancer was High‐Grade Serous Ovarian Cancer. However, High‐Grade Serous Ovarian Cancer organoid is rare, which organoid with patient immune microenvironment and blood vessels even absence. Here, we report a novel High‐Grade Serous Ovarian Cancer organoid system derived from patient ovarian cancer samples. These organoids recapitulate High‐Grade Serous Ovarian Cancer organoids' histological and molecular heterogeneity while preserving the critical immune microenvironment and blood vessels, as evidenced by the presence of *CD34* ^+^ endothelial cells. Whole exome sequencing identifies key mutations (*CSMD3*, *TP53*, *GABRA6*). Organoids show promise in testing cisplatin sensitivity for patients resistant to carboplatin and paclitaxel, with notable responses in cancer proteoglycans and *p53* (*TP53*) signaling, like *ACTG*/*ACTB1*/*AKT2* genes and *BBC3*/*MDM2*/*PERP*. Integration of immune microenvironment and blood vessels enhances potential for novel therapies like immunotherapies and angiogenesis inhibitors. Our work may provide a new detection system and theoretical basis for ovarian cancer research and individual therapy.

## Graphical abstract

We have developed a matrigel and enzyme‐free organoid culture method for High‐Grade Serous Ovarian Cancer, enabling accurate representation of the tumor microenvironment and vascularization features. This approach facilitates a comprehensive understanding of the cellular composition and internal characteristics of ovarian cancer, while also demonstrating its potential in rapid drug screening and selection for clinical applications.

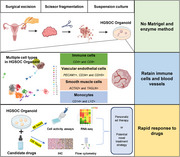

## 1 INTRODUCTION

Ovarian cancer, a complex and multifaceted disease, remains a formidable challenge in oncology, with High‐Grade Serous Ovarian Cancer (HGSOC) being the most aggressive and prevalent form.^1^, ^2^ Despite advances in detection and treatment, HGSOC is characterized by high recurrence, incidence, and mortality rates that underscore the urgent need for more effective therapeutic strategies.^3^, ^4^, ^5^ The 5‐year relative survival rate for all types of ovarian cancer is approximately 50%, with significant variation depending on factors such as the stage at diagnosis, age, and health of the patient, and the efficacy of the treatment plan.^6^, ^7^, ^8^ The standard first‐line treatment for ovarian cancer postsurgery has been a regimen of platinum‐based chemotherapy, such as carboplatin or cisplatin, combined with paclitaxel or similar drugs.^9^, ^10^ While this approach can be initially effective, approximately 70% of HGSOC patients experience a recurrence, highlighting the limitations of current therapies and the critical need for alternative strategies.^11^, ^12^, ^13^, ^14^ In cases of platinum‐resistant ovarian cancer, a variety of treatments are considered, including targeted therapy with Bevacizumab, which inhibits angiogenesis by blocking VEGF‐A, and PARP inhibitors like Niraparib and Olaparib that interfere with DNA repair mechanisms in cancer cells.^11^, ^15^, ^16^ Additionally, the integration of chemotherapy with immunotherapies, such as checkpoint inhibitors Nivolumab and Pembrolizumab, represents a promising avenue of exploration.^17^

HGSOC, characterized by its aggressive nature and high relapse rate, exemplifies the limitations of a one‐size‐fits‐all treatment approach. This situation highlights a profound need for personalized treatment plans, driven by a deep understanding of individual tumor biology and patient‐specific response patterns to various therapies.^18^, ^19^ The intricate interplay of genetic, molecular, and environmental factors influencing tumor behavior necessitates a departure from traditional treatment modalities towards more individualized strategies.^1^, ^20^, ^21^, ^22^ The advent of organoid technology, which allows for the cultivation of patient‐derived tumor cells in three‐dimensional structures that closely replicate the cellular heterogeneity, genomic characteristics, and microenvironment of the original tumor, offers a groundbreaking approach to precision medicine.^23^, ^24^, ^25^, ^26^ By enabling high‐fidelity disease modeling and drug response testing, organoids hold the potential to transform the landscape of ovarian cancer treatment, paving the way for highly individualized and effective therapeutic strategies. This approach not only enhances the efficacy of treatment but also minimizes the risk of adverse effects and the development of resistance.^27^, ^28^, ^29^, ^30^, ^31^

In the evolving landscape of ovarian cancer research, the development of models that accurately reflect the complex biology of HGSOC remains a critical challenge.^32^ Herein, we introduce a meticulously developed method for the swift creation of ovarian cancer organoids from freshly procured tumor samples, employing a defined culture medium without necessitating single‐cell dissociation. Our initiative has led to the establishment of a little biobank of ovarian cancer organoids, alongside thorough histological, molecular, and genomic evaluations demonstrating that these organoids closely mirror the heterogeneity observed both between and within tumors. These organoids faithfully preserve key features of their parental tumors, including specific immune cell markers and vascular endothelial markers, thus demonstrating the advanced methodological design and biological fidelity of the organoid platform.

Our organoids have been meticulously engineered to encapsulate a wide array of cellular markers identified through single‐cell RNA sequencing (scRNA‐seq), providing insight into the cellular heterogeneity inherent to HGSOC. This includes the identification of specific immune cell markers and vascular endothelial markers, crucial for understanding the interactions within the tumor microenvironment. For instance, the presence of *CD4, CD8A*, and *CD8B* for T cells, and *PECAM1*, *CD34*, and *CDH5* for endothelial cells within our organoids offers a nuanced view of the tumor's immune landscape and vascular network. This comprehensive characterization exemplifies our dedication to developing a model that closely mimics the complex biological landscape of ovarian malignancies. A cornerstone of our research has been the application of these organoids in assessing the responsiveness to cisplatin, particularly for patients who have previously undergone treatment with carboplatin and paclitaxel. This approach yielded insights that are closely aligned with the patients’ individual reactions, thereby offering a predictive framework for addressing resistance to standard chemotherapy regimens. It is a testament to our organoids’ utility in facilitating personalized treatment strategies, marking a stride towards precision oncology.

## 2 RESULTS

### 2.1 Culture and characterization of ovarian cancer organoids from patient‐derived tumors

**FIGURE 1:**
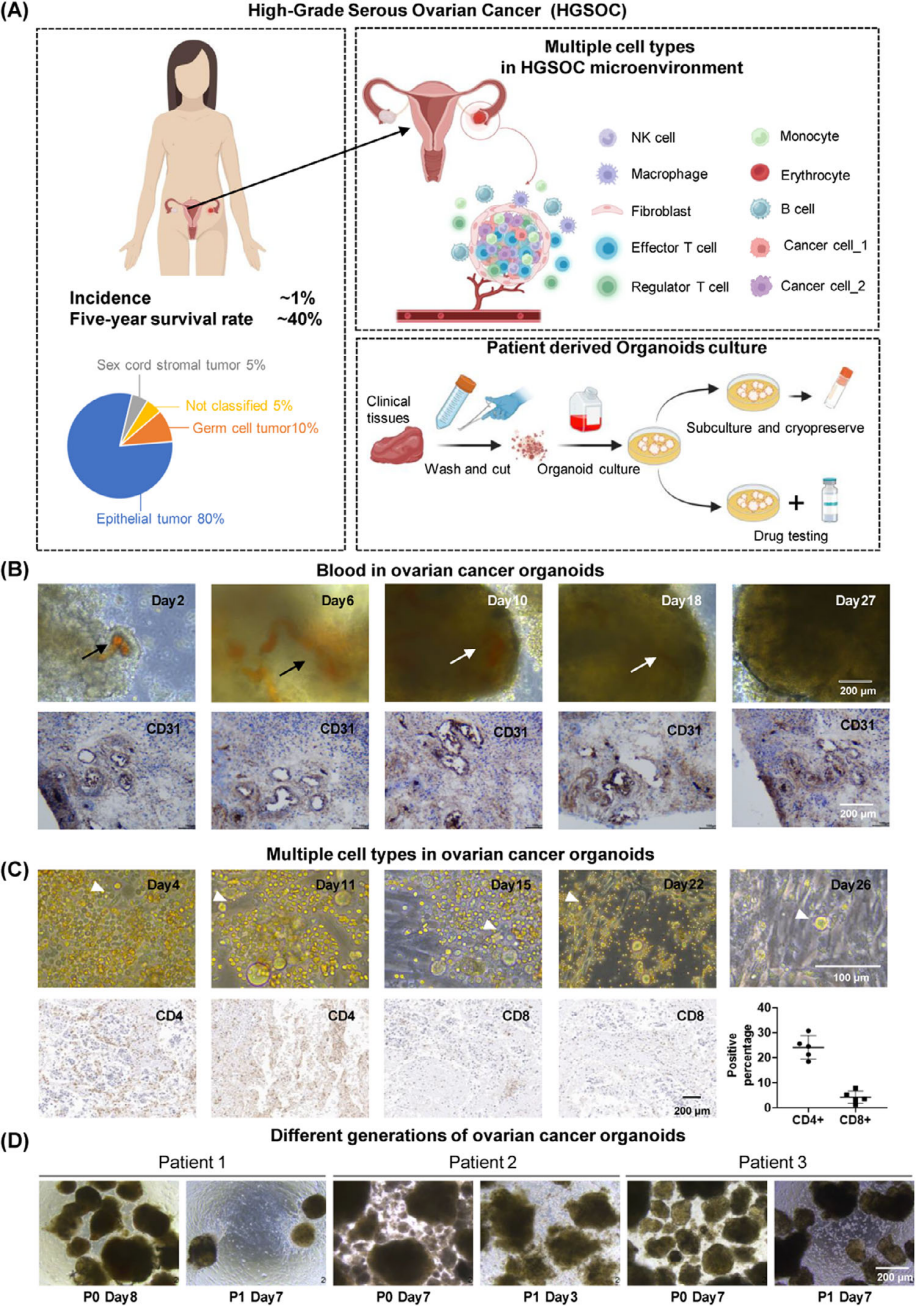
Establishment of reproducible ovarian cancer organoid model. The tumor tissue obtained from the patient was washed and minced into pieces of less than 2 mm in diameter using dissecting scissors. The minced tissue was then resuspended in culture medium and culture in Petri dish. (A) Left: Overview of HGSOC. Right: Cell types in HGSOC and schematic diagram of establishment of ovarian cancer organoids. Throughout the culture process, neither enzymatic digestion nor matrigel were employed. (B) Top: Morphology of P0 ovarian cancer organoids. Blood vessels were visible until day 18. Arrows indicate blood vessels in ovarian cancer organoids. Bottom: Distribution of CD31 positive cells in organoids. The tubular distribution indicated existence of blood vessels. Different images originate from different sections of the same organoid. (C) Cells in P0 ovarian cancer organoids. Top: In addition to cells evolved in organoids, suspension cells with diversity morphology were maintained in culture system. Tips point to different cell types in dish. Bottom: The presence of CD4^+^ and CD8^+^ T cells within the organoids, as demonstrated by IHC staining. (D) Subculture of ovarian cancer organoids. Ovarian cancer organoids maintained growth ability from P0 to P1.

In an effort to closely emulate the intricate morphological characteristics and crucial cell–cell interactions present in original ovarian tumors, we devised a novel organoid culture protocol. This method circumvents the enzymatic dissociation of tumors into single cells, thereby preserving the structural integrity and cellular heterogeneity of the original tumor tissue. We totally established 7 organoids in this research, including OV004 (61 years old, HGSOC), OV014 (68 years old, HGSOC), OV015 (52 years old, HGSOC), OV016 (69 years old, HGSOC), OV076 (59 years old, right‐sided fallopian tube mass), OV077 (96 years old, unknown) and OV078 (67 years old, unknown). Utilizing precision‐cutting techniques, we sectioned patient‐derived ovarian tumors into fragments approximately 1 mm in size. These fragments were sourced from various regions within the tumors to encompass a broad representation of the tumor's cellular composition (Figure 1A). Notably, our protocol deliberately abstained from removing red blood cells and tissue debris, a step we hypothesized would aid in maintaining the organoid's fidelity to the tumor's microenvironment.

Upon incubation at standard culture conditions, we observed the formation of three distinct ovarian cancer organoids. Each organoid exhibited successful culture establishment and, within approximately 2 weeks, demonstrated spherical growth, indicative of their proliferative capacity. Histological examination of the organoids revealed the preservation of key features characteristic of ovarian tumors, including the presence of intra‐organoid vascular structures. Interestingly, despite our method not incorporating a red blood cell removal step, the vascular features within the ovarian cancer organoids were distinctly observable, with blood vessels persisting for up to 18 days in culture (Figure 1B). Immunohistochemical analysis further substantiated the presence of vascular structures, with CD31 expression delineating a tubular vascular pattern within the organoids (Figure 1B). Moreover, our culture conditions facilitated the preservation of diverse cell types, suggesting the retention of the cellular diversity within the organoids, especially CD4+ and CD8+ T cells (Figure 1C).

The ovarian cancer organoids’ capacity for subculture was also evaluated. Following a 28‐day incubation period, the organoids were mechanically divided and redistributed across new culture vessels. Remarkably, these secondary organoids mirrored the initial spherical morphology observed in their predecessors, although they lacked vascular components, likely attributable to the absence of hematopoietic functionality (Figure 1D).

Our study demonstrates the successful establishment of ovarian cancer organoids that not only maintain growth ability but also show potential in preserving the native immune microenvironment and blood vessel. This advancement represents a significant step forward in the development of biologically relevant in vitro models for ovarian cancer, offering promising avenues for future therapeutic research and personalized medicine approaches.

### 2.2 Preservation of tumor molecular characteristics in ovarian cancer organoids

**FIGURE 2:**
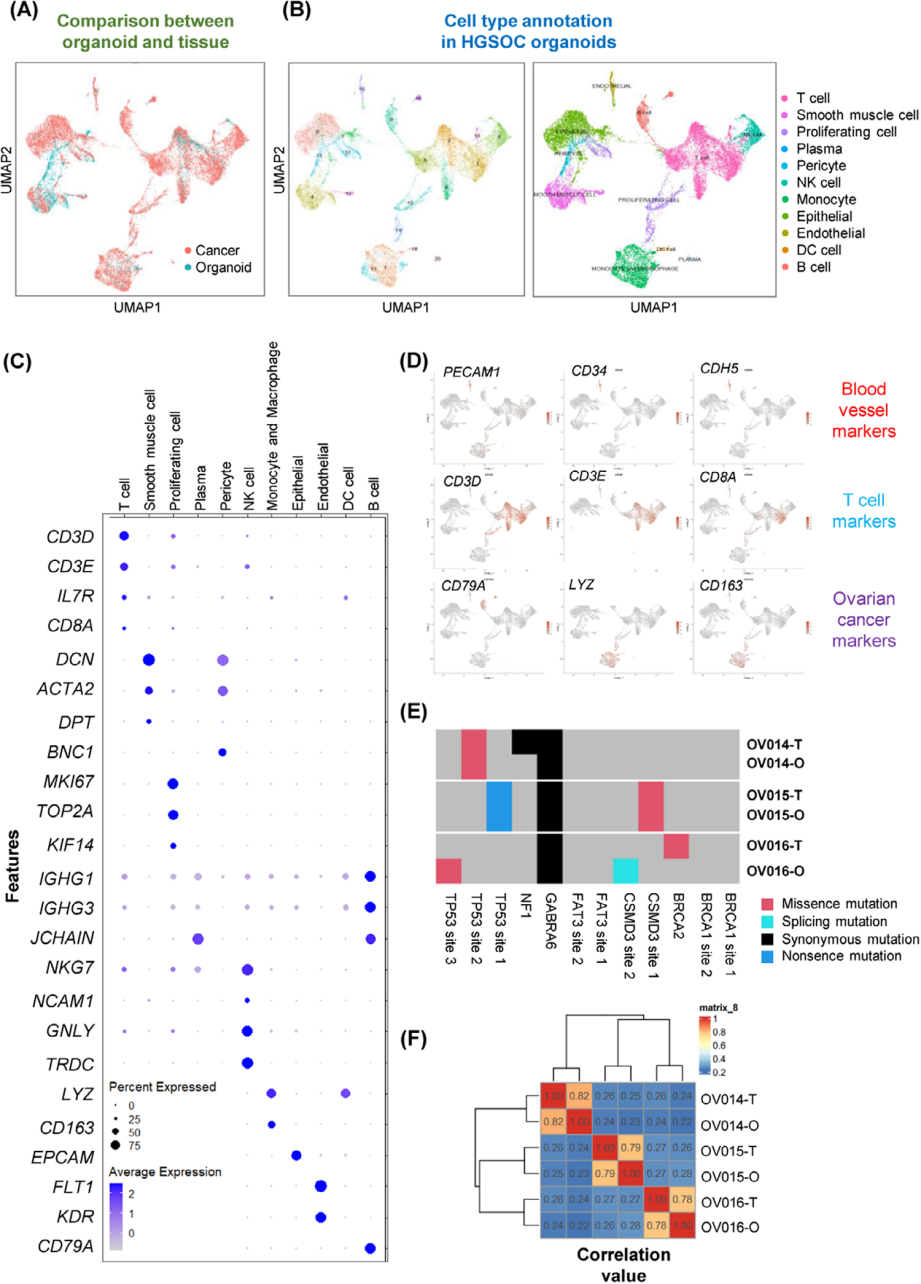
Results of scRNA‐seq and WES of ovarian organoids. (A) UMAP visualization of single‐cell RNA‐seq results compared with public dataset. The distribution patterns of UMAP of organoids and tissues exhibit similarity. (B) Annotation of organoids and ovarian cancers regarding the composition of cell types. All 11 cell types were identified in both organoids and tissues, demonstrating the similarity between ovarian cancer organoids and tissues. (C) Expression profiles of different cell type marker genes in organoids and cancer tissues. (D) Marker genes of blood vessel cells, T cells, and other immune cells. Top: Blood vessel cell marker genes *PECAM1*, *CD34*, and *CDH5* expressed in endothelial cells, indicating the presence of vascular endothelial cells. Middle: Three clusters of T cells were annotated into CD3^+^ and CD8^+^ T cells. Bottom: Marker genes of B cells (*CD79A*), macrophages (*CD163*), and monocytes (*LYZ*). (E) Mutation types of critical cancer genes in tissue and organoids, including *BRCA1/2*, *TP53*, and *CSMD3*. (F) Correlation value between samples, including tissues and organoids. Organoids and its parent tissues maintain high correlation value compared with other groups.

Addressing the inherent challenge of replicating the complex heterogeneity of ovarian tumors, our study focuses on the capacity of ovarian cancer organoids to mirror the diverse cellular makeup of the parent tumors accurately. Employing scRNA‐seq at day 14 postformation, we identified distinct cell clusters within the ovarian cancer organoids. Cross‐referencing with recent single‐cell sequencing datasets from ovarian cancer tissues, the cellular composition of our organoids is largely similar to that of public data (Figure 2A and B). These included T cells (positive for *CD3* and *CD8*), endothelial cells (positive for *CD34* and *FLT1*), smooth muscle cells (*DCN* and *ACTA2* positive), monocytes and macrophages (*LYZ* and *CD163* positive), and B cells (*CD79A* positive). This evidence confirms the existence of equivalent cell populations within the ovarian cancer organoids, validating the capacity of our organoid model to faithfully recapitulate the cellular diversity inherent to ovarian tumors (Figure 2B).^33^

Focusing on the organoids’ microenvironment, particularly the vascular and immune components, we analyzed the expression of key marker genes. Notably, the presence of vascular endothelial markers, such as *FLT1*, *CD34*, and *KDR*, underscores the organoids’ capability to maintain blood vessel‐like structures. Simultaneously, the detection of T‐cell markers, including *CD3* and *CD8*, highlights the preservation of immune cell subsets within the ovarian cancer organoids. This cellular composition, closely mirroring that of the original ovarian tissues, emphasizes the ovarian cancer organoids’ utility as a robust model for studying ovarian cancer in a physiologically relevant context (Figure 2C and D).

To assess the genetic stability of ovarian cancer organoids over the culture period, whole exome sequencing (WES) was performed, revealing the preservation of key ovarian cancer‐related mutations, including *CSMD3*, *TP53*, and *GABRA6*. Comparative analysis between the organoids and their tissue of origin demonstrated a high correlation, affirming that ovarian cancer organoids maintain the genetic landscape of the parent tumors throughout the culture process (Figure 2E and F).

Our findings underscore the ovarian cancer organoids’ remarkable ability to preserve the complex cellular and molecular landscape of ovarian tumors, offering a promising platform for in‐depth studies on tumor biology, drug testing, and personalized medicine approaches in ovarian cancer.

### 2.3 Preservation of tumor pathological phenotypes in ovarian cancer organoids

**FIGURE 3:**
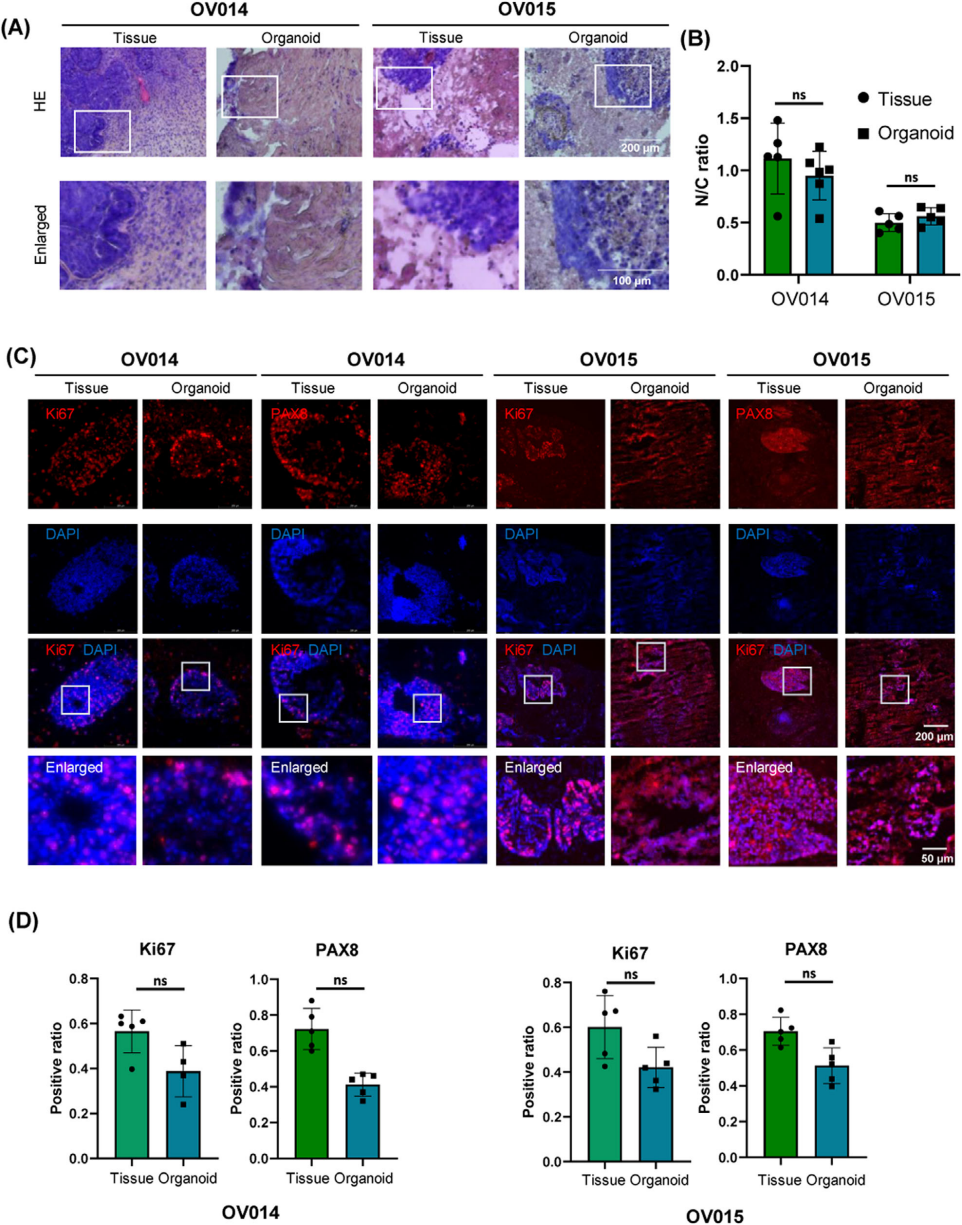
Resemblance of histopathological characteristics and biomarker expression patterns between ovarian cancer organoids and primary tissues. All image data were derived from P0 organoids. (A) Similar structures between tissues and organoids stained with H&E. (B) Organoids maintained similar N/C ratio compared with tissues, indicated its cancerous characteristic. The ratio of nuclei and cytoplasm were defined by H&E staining area. (C) ICC results of ovarian cancer organoids and tissues indicated similar distribution patterns of Ki67 and PAX8 in organoids and tissues. (D) Expression level of cell markers PAX8 and Ki67. Expression level of Ki67 was similar between organoids and tissues. However, expression of PAX8 decreased in organoids, possibly due to in vitro culture environment. ns, no significance.

The criticality of maintaining not only cellular types but also their inherent organizational architecture to emulate biological function is paramount in our study. Our assessment of the organoids' pathological features elucidates this cellular organization and the expression profiles of pivotal biomarkers. Utilizing hematoxylin and eosin (H&E) staining, we established a clear parallelism in the tissue architecture between the organoids and their clinical counterparts (Figure 3A), affirming the organoids' capability to conserve the structural integrity observed in vivo. Additionally, the nuclear‐to‐cytoplasmic (N/C) ratio, a metric indicative of malignancy, was closely studied. The comparative N/C ratios, 1.056 in tissue versus 0.901 in organoids for one patient pair and 0.524 versus 0.578 for another, demonstrate the organoids' proficiency in mirroring the cytological features pertinent to cancer assessment (Figure 3B).

In the clinical setting, Ki67 serves as a prognostic marker for ovarian cancer, reflecting cellular proliferation rates, while PAX8 identifies the gynecological origin of the malignancy, correlating with patient outcomes. Our meticulous immunohistochemical (IHC) evaluation demonstrated analogous expression and localization patterns for Ki67 and PAX8 across organoids and tissue samples (Figure 3C). Despite some quantitative differences—38.8–42.1% for Ki67 and 41.2–51.3% for PAX8 in organoids versus 70.5–72.2% and 56.5–60.1% in tissues, respectively (Figure 3D)—these variations provide insights into the microenvironmental adaptation within the organoid culture.

Collectively, these findings substantiate the organoids' utility in preserving and reflecting the pathological nuances of ovarian cancer, showcasing their potential as an instrumental model for the pathological study and therapeutic assessment of this disease. Through these organoid models, we can achieve a more nuanced understanding of ovarian cancer's pathology, facilitating advanced research and personalized treatment approaches.

### 2.4 Enhanced cisplatin sensitivity assessment through patient‐derived ovarian cancer organoid models

**FIGURE 4:**
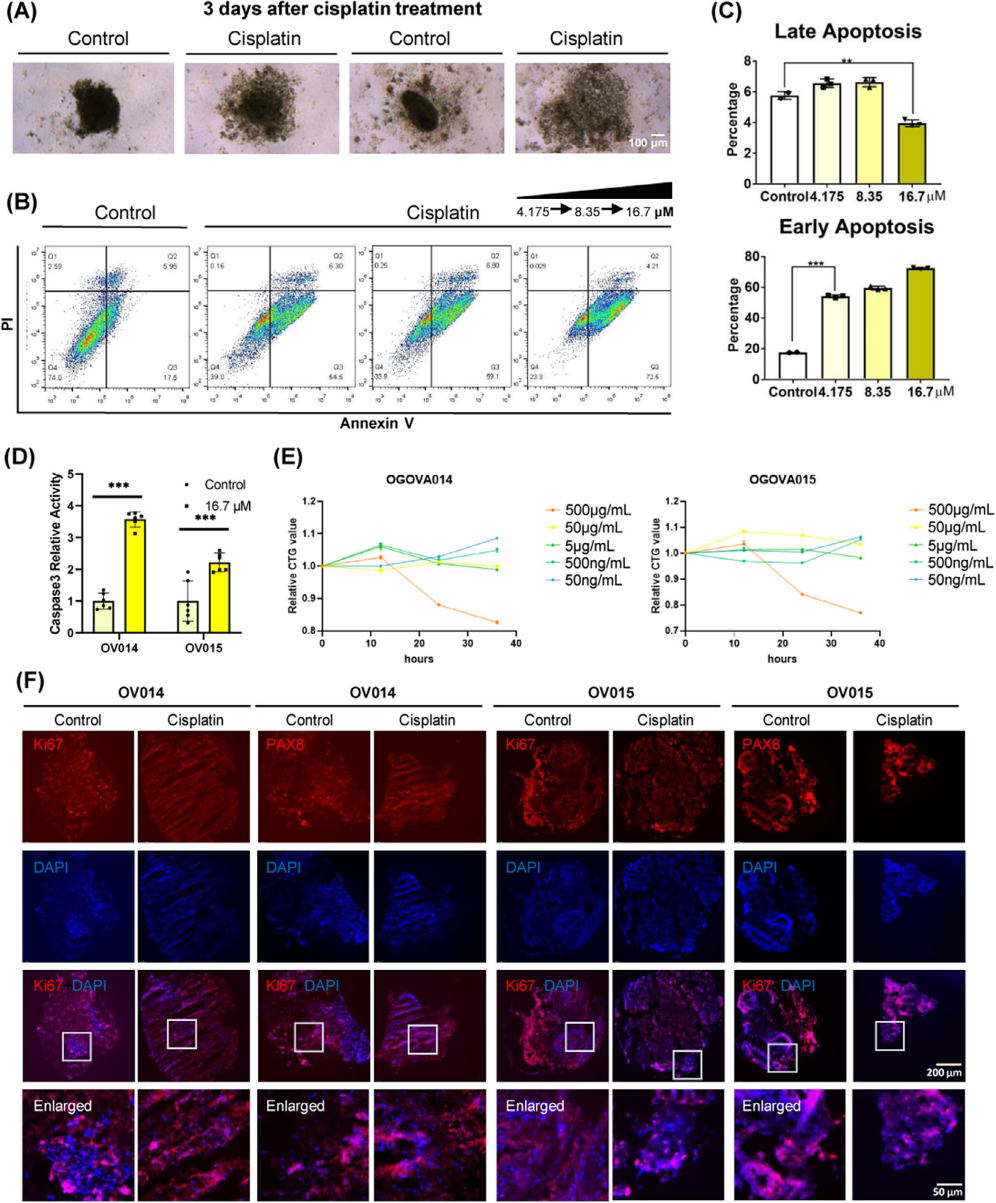
Cisplatin sensitivity test using ovarian cancer organoids. (A) Morphology changes between control and cisplatin‐treated organoids. After cisplatin treatment, ovarian cancer organoids became obvious collapse. (B, C) Early‐stage apoptosis was induced by cisplatin treatment validated with flow cytometry. (D) Caspase3, a biomarker of early‐stage apoptosis, increased after cisplatin treatment, validating that early‐stage apoptosis occurs. (E) CTG assay results of organoids under different concentration cisplatin treatment. (F) Expression level of Ki67 decreased after cisplatin treatment, but PAX8 maintained a stable level. **p < 0.01; ***p < 0.001.

Central to our investigation has been the application of patient‐derived ovarian cancer organoids for evaluating cisplatin responsiveness, particularly in the context of patients previously treated with carboplatin and paclitaxel. To establish a rapid screening method for drug sensitivity, we assessed the impact of cisplatin on ovarian cancer organoids. Notably, after a 3‐day exposure to 16.7 µM of cisplatin, the treated organoids exhibited a marked structural collapse compared to the untreated control group, suggesting significant sensitivity to cisplatin at this concentration (Figure 4A). Further elucidation of cisplatin's effect was obtained through flow cytometry analysis of cell apoptosis. This revealed a substantial increase in the fraction of early‐stage apoptotic cells (PI‐/Annexin V+) posttreatment, with the extent of early‐stage apoptosis amplifying in response to higher cisplatin concentrations. Conversely, the proportion of cells in late‐stage apoptosis remained unchanged, underscoring cisplatin's mechanism of action through the induction of early apoptosis (Figures 4B and C). Corroborating this, caspase‐3 levels, indicative of early‐stage apoptosis, were significantly elevated in cisplatin‐treated cells (Figure 4D). This dose‐dependent effect highlights the ineffectiveness of low cisplatin concentrations in suppressing ovarian cancer organoids’ activity, emphasizing the drug's cytotoxic efficacy at higher doses. Indeed, different concentrations of cisplatin have different inhibitory effects on organoids (Figure 4E). Immunocytochemistry (ICC) analysis further demonstrated a diminished proliferation capacity within the organoids following cisplatin treatment, as evidenced by a significant reduction in Ki67 expression. In contrast, PAX8 expression remained unaffected, indicating the specificity of cisplatin's action on proliferative markers (Figure 4F).

Collectively, our findings affirm that cisplatin significantly curtails the proliferative capabilities of ovarian cancer organoids, aligning with clinical observations and underscoring the utility of ovarian cancer organoids in devising personalized treatment plans. This study also reinforces the predictive value of organoids in fast‐assessing drug sensitivity.

### 2.5 Deepened molecular understanding of cisplatin responsiveness through patient‐derived ovarian cancer organoid models

**FIGURE 5:**
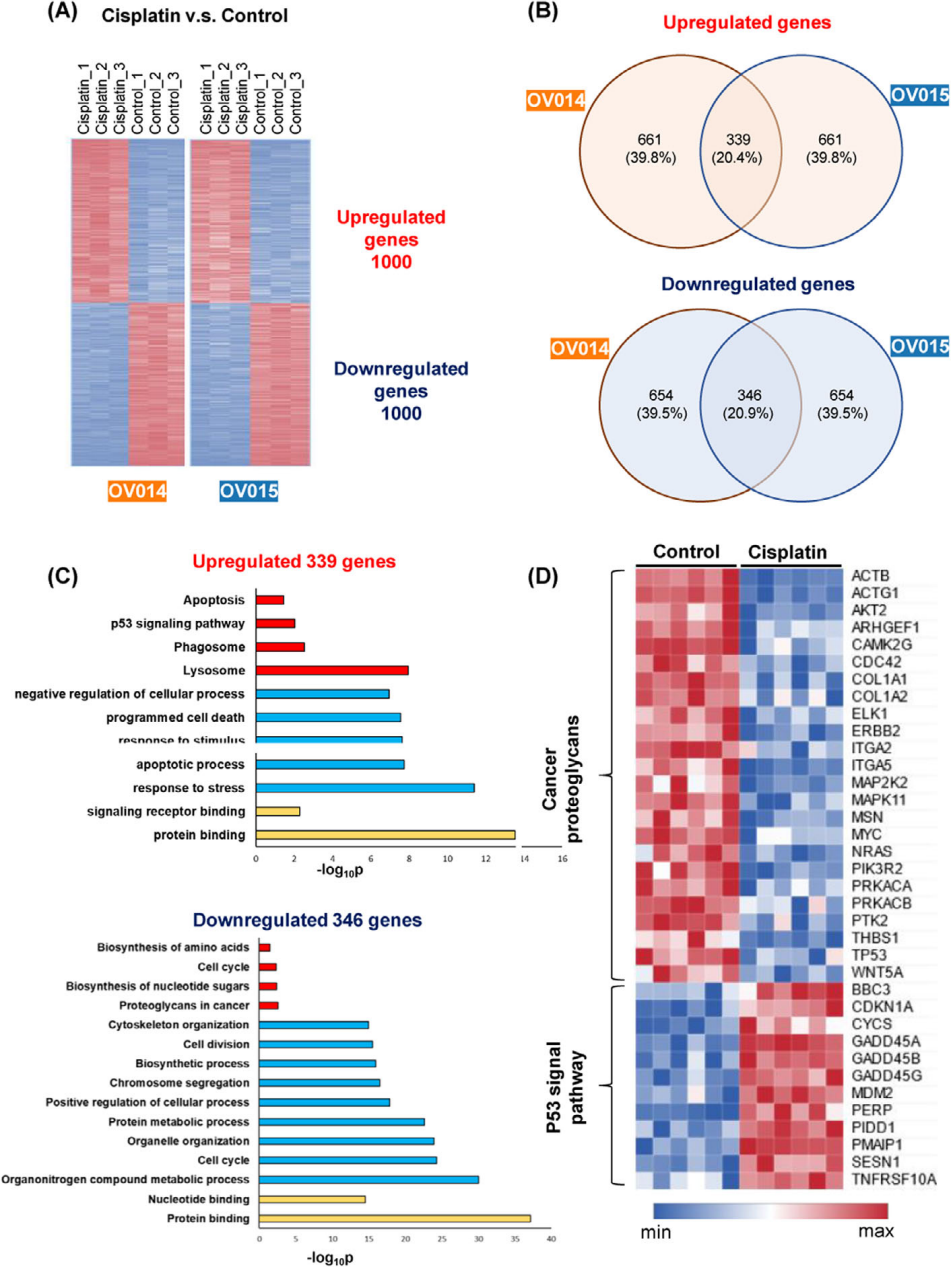
Transcriptome analysis of cisplatin‐treated ovarian cancer organoids. (A) Obvious changes in gene expression profiles between control and cisplatin‐treated organoids. Only top 1000 genes that were up‐ or downregulated are shown. (B) Organoids derived from two individual patients showed an overlap of regulated genes. Despite heterogeneity between patients, similar response occurred in two organoid lines. (C) GO and KEGG analysis of regulated genes in two organoid lines. Consistent with previous studies, p53 pathway was upregulated under cisplatin treatment. (D) Heatmap of genes in p53 pathway and cancer proteoglycans synthesis.

In order to study the intricacies of cisplatin's impact on ovarian cancer at the molecular level, we conducted RNA‐seq analysis on two sets of patient‐derived ovarian cancer organoids subjected to cisplatin treatment. The analysis revealed significant alterations in the gene expression profiles under the influence of cisplatin, demonstrating the drug's pervasive action across various patient‐derived models (Figure 5A). Among the top 1000 genes modulated by cisplatin treatment, there was a substantial overlap, with 339 genes upregulated and 346 genes downregulated in both sets of organoids (Figure 5B). This overlap highlights the consistent and broad‐spectrum efficacy of cisplatin across different ovarian cancer backgrounds.

Further exploration of these changes through Gene Ontology (GO) and Kyoto Encyclopedia of Genes and Genomes (KEGG) pathway analyses pinpointed specific clusters of genes that were differentially regulated by cisplatin treatment. Notably, there was an enhanced expression of genes associated with apoptosis in the cisplatin‐treated organoids, in contrast to the control group, which showed elevated expression of genes involved in cell division (Figure 5C). This differential gene expression pattern aligns with our understanding that cisplatin exerts its anticancer effects primarily through the induction of apoptosis.

Particularly, the analysis shed light on the differential pathway activation between the control and cisplatin‐treated groups (Figure 5D). Control organoids demonstrated heightened activity of genes related to cancer proteoglycans, indicating an active metabolic state conducive to cancer progression. Conversely, the cisplatin‐treated organoids exhibited a marked upregulation of genes within the p53 signaling pathway, suggesting that cisplatin's mechanism of inhibiting cancer cell proliferation is mediated through apoptosis, triggered by the activation of the p53 pathway. This observation is in harmony with previous study,^34^, ^35^ underscoring the pivotal role of the p53 pathway in cisplatin‐induced apoptosis mechanisms. Moreover, our RNA‐seq analysis unveiled a notable reduction in the expression of genes crucial for cell proliferation and tumor genesis, such as *Ki67* and *BRCA1/2*, following cisplatin administration. This finding substantiates the drug's efficacy in curtailing oncogenic processes within the organoids.

In summary, notwithstanding the interpatient heterogeneity, the organoid models consistently recapitulated the clinical efficacy of cisplatin, corroborating the notion that these organoids retain patient‐specific biological features and substantiating their value in drug sensitivity testing. This convergence of clinical and organoid‐based responses to cisplatin underscores the potential of organoid models in facilitating personalized oncology, particularly in predicting and understanding patient‐specific responses to chemotherapy.

## 3 DISCUSSION

This study introduces a novel organoid model derived from patient ovarian tumors, encapsulating the complex heterogeneity and microenvironmental intricacies of HGSOC. Our findings underscore the organoids’ remarkable fidelity in replicating the histological and molecular landscapes of the originating tumors, alongside preserving key components of the immune system and vascular structures. This mirrors the methodological advancements and contributions to the field of cancer research akin to those detailed in our reference document, emphasizing the organoid model's potential in translational research.

Ovarian cancer is characterized by its significant heterogeneity and a commonality of late‐stage diagnosis, presenting substantial challenges in both understanding and treating this malignancy effectively.^36^ Despite huge advancements,^2^, ^3^, ^4^ the field of ovarian cancer organoid modeling still faces considerable hurdles, particularly in the accurate simulation of tumor markers and the cancer's inherent heterogeneity.^37^ These aspects are crucial for a realistic representation of the disease, yet existing models often fall short in capturing this complexity fully. A more nuanced organoid model that endeavors to closely approximate the tumor markers and heterogeneity inherent in ovarian cancer could potentially facilitate advancements in critical areas of research.

A principal advantage of our approach lies in the maintenance of the immune microenvironment and vasculature within the organoids, alongside the rapid assessment of drug responses. This integration of immune and vascular markers signifies a critical methodological progression, enabling a more detailed evaluation of therapeutic effectiveness and the mechanisms of resistance. These advancements also deepen our comprehension of the biological intricacies of HGSOC.

While our study represents a significant step forward in ovarian cancer research, it is not without limitations. The scalability of organoid models and the potential for selection bias in tumor sample procurement necessitate cautious interpretation of our results. However, the strengths of our study, particularly the methodological innovations in organoid development and the comprehensive analysis of chemotherapy responsiveness, provide a solid foundation for future research. Our previous research has demonstrated that our organoid model also responds to PARP inhibitors, suggesting the potential of this model in drug sensitivity testing for various types of medications.^38^

Looking ahead, the potential applications of our organoid model extend beyond the scope of this study. Using the aforementioned experimental methods, we have established organoid models including dermatofibrosarcoma protuberans (DFSP), intrahepatic cholangiocarcinoma (ICC), and glioblastoma.^39^, ^40^, ^41^, ^42^, ^43^ Future research should explore the organoids' utility in screening a broader range of therapeutic agents, including immunotherapies and targeted therapies, to further tailor treatment plans to individual patient profiles. Additionally, expanding the organoid model to encompass other cancer types could offer new avenues for understanding and treating cancer at a personalized level.^44^

## 4 METHODS

### 4.1 Patient information

Ovarian cancer tissues were collected from 7 individual patients (OV004(61 years old, HGSOC), OV014(68 years old, HGSOC), OV015(52 years old, HGSOC), OV016(69 years old, HGSOC), OV076(59 years old, Right‐sided fallopian tube mass), OV077(96 years old, unknown) and OV078(67 years old, unknown)) with ovarian cancer at Obstetrics and Gynecology Hospital of Fudan University in 2022, after obtaining informed consent of the patient before surgery. OV004 was used for scRNA‐seq. OV014, OV015, and OV016 were used for living microscopy observe and subsequent experiments. OV014 and OV015 were used for cisplatin treatment, ICC staining and RNA‐seq. OV076, OV077, and OV078 were utilized for live microscopic observation. These patients underwent a laparotomy primary debulking surgery with optimal cytoreduction. Our study was approved by the Ethics Committee of the Obstetrics and Gynecology Hospital of Fudan University.

### 4.2 Organoids formation, culture, and subculture

Patient‐derived OC tissues were washed 4−6 times with organoid washing buffer (LSTO00100201, Shanghai Lisheng Biotech, China) and then dissociated to about 1 mm diameter pieces with surgical scissors, no collagenase, trypsin or dispase needed. Tumor pieces were resuspended ovarian cancer organoid medium (LSTO00100501, Shanghai Lisheng Biotech, China) and transferred into new dishes. The ovarian organoids were maintained with at 37°C, 5% CO_2_, and medium was semichanged per week. When the diameter of organoids reached 2 to 4 mm (about 4 weeks), the organoids were collected and mechanically dissociated, then subcultured as described above.

### 4.3 Whole exome sequencing

For each sample, 200 ng genomic DNA was sheared to 150−200 bp fragments to construct libraries. The whole exome was captured using AIExome® Human Exome Panel V3 with TargetSeq One® Hyb & Wash Kit v2.0 (iGeneTech Co., Ltd, Beijing, China) and sequenced on DNBSEQ‐T7 with 150‐bp reads.

### 4.4 Immunofluorescence and immunohistochemistry

Tissue or organoids were first fixed with 4% PFA (BL539A, Biosharp, China), then dehydrated with 10%, 20% and 30% sucrose solution sequentially. Embedding medium with samples (7.5% gelatin and 10% sucrose in 1× PBS) was cooled at 4°C for 10 min and quickly frozen on dry ice for 10 min, then stored at −80°C. Materials were sectioned using Leica CM 1950. On‐plate adherent cells were fixed with 4% PFA for 20 min at room temperature. Permeabilization of cells and sections was performed with PBS+0.25% TritonX‐100 for 18 min at room temperature, followed by blocking with 3% BSA.

For immunofluorescence, rabbit anti‐Ki67 (1:1000; MA5‐14520, ThermoFisher, England) and anti‐PAX8 (1:1000; 10336‐1‐AP, Proteintech, China) were used as primary antibodies to stain proteins overnight at 4°C. Samples were then washed 5 times with 0.125% PBST and incubated with the CY3‐anti‐rabbit secondary antibody (1:1000) for 3 h at room temperature. Nuclei were stained by DAPI (1:1000).

For immunohistochemistry, mouse anti‐CD31 (1:250; ab9498, Abcam, USA) were used as primary antibodies to stain CD31 positive cells. Samples were incubated overnight at 4°C then washed 5 times with 0.125% PBST and incubated with the HRP‐anti‐mouse secondary antibody (1:500; ab6789, Abcam, USA) for 3 h at room temperature. Recolor the slices using DAB substrate kit (ab64328, Abcam, USA).

### 4.5 RNA sequencing

For bulk RNA, RNA Isolation Kit (DP451, TIANGEN, China) was used to isolate cell mRNA. Four to six 2‐mm‐diameter organoids were collected as input for each group of organoids. Concentration of RNA was measured using Qubit4 fluorometer. Reverse transcription was performed with RT Kit (KR118, TIANGEN, China) and libraries were generated with RNA Library Prep Kit (E7530L, NEB, USA).

For single cell RNA, libraries were generated with 10× genomics protocol using 15,000 cells as input. Both libraries were sequenced on the Illumina Hiseq 4000 with PE‐150 bp reads for subsequent analysis.

### 4.6 Bioinformatics analysis

For RNA‐seq, the kallisto software was utilized to quantify transcript abundance, employing Fragments Per Kilobase of transcript per Million mapped reads to estimate RNA expression levels. Differentially expressed genes were identified using the Morpheus online platform. In the case of scRNA‐seq, the raw data were processed using Cell Ranger, with subsequent analyses conducted via the Seurat package. For WES, the BWA‐MEM algorithm facilitated alignment of raw sequence data to the reference human genome (hg38). Downstream analyses, including variant calling, were performed using the Genome Analysis Toolkit and Mutect2.

### 4.7 Cisplatin sensitivity test

Organoids were cultured in 6‐well plates as mentioned above, and cisplatin was added into medium at 8/48 h after the initial time point at final concentrations of 4.175, 8.35, and 16.7 µM. At 168 h, the organoids were collected and sectioned as described above.

### 4.8 Microscopy observing

All bright‐field images were taken using Leica Ti‐1 microscope. Fluorescence images were taken using Leica K5 microscope.

### 4.9 Caspase3 activity assay

Organoids cultured with protocols described above were dissociated with 0.25% trypsin and then cultured in 96‐well ultralow attachment plates at 10,000 to 20,000 cells per well. Cisplatin was added to concentration of 16.7 µM, and caspase3 activity assay was performed using Promega G8091 kit after 3 days treatment.

### 4.10 CTG assay

Organoids were cultured in 6‐well plates as mentioned above, and cisplatin was added into medium at the beginning of culture. At 0, 12, 24, and 36 h respectively, same amount of organoids were collected and performed CTG assay with CellTiter‐Glo Luminescent Cell Viability Assay Kit (G7572, Promega).

### 4.11 Cell cytometry

Organoids were dissociated with collagenase NB 4 in 0.25% trypsin, then collect 1 × 10^6^ cells in 1.5 mL EP tube and centrifuge at 250 × *g* for 3 min. Cells were suspended in 1× PBS and stained with PI (0.01 mg/mL) and annexin V (1:100) for 15 min. Cells were then centrifuged at 250 × *g* for 3 min and collected. The cell flow cytometry was performed with a BD FACS Verse kit (Franklin Lakes, NJ, USA) following standard procedures.

### 4.12 Statistical analysis

In ICC assay and qPCR, data analysis was performed using Prism software. *p* Value < 0.05 was considered statistically significant. All data are expressed as mean ± standard deviation.

## AUTHOR CONTRIBUTIONS

HXX, CCH, and CQ conceived and designed the experiments; ZYQ, WC, LLY, SYH, and ZJ performed the experiments; ZYQ, WC, JPL, and CCH analyzed the data; TX gave pathology analysis of ovarian cancer; ZYQ, WC, DW, CCH, and HXX wrote the manuscript. All the authors approved the final manuscript.

## CONFLICT OF INTEREST STATEMENT

Author Chen Wang, Xinxin Han, Jian Zhang, Chunhui Cai are the employees in Shanghai LiSheng Biotech, but has no potential relevant financial or non‐financial interests to disclose. The other authors have no conflicts of interest to declare.

## ETHICS STATEMENT

This study was approved by the Ethics Committee of the Obstetrics and Gynecology Hospital of Fudan University (kyy2023‐06). Written informed consent was obtained from all participants. All human specimens were performed according to the Declaration of Helsinki and the Chinese guidelines of GB/T4352.1‐2021 and GB/T38736‐2020. Approved by the Ethics Committee of the Obstetrics and Gynecology Hospital of Fudan University, patients/subjects signed a written informed consent. All human tissues were collected with patient consent according to clinical SOPs.

## Data Availability

The raw sequence data reported in this paper, including Bulk‐RNAseq, sc‐RNAseq, and WES, have been deposited in the Genome Sequence Archive (GSA) at the National Genomics Data Center, Beijing Institute of Genomics, Chinese Academy of Sciences. The data are publicly accessible at https://ngdc.cncb.ac.cn/gsa‐human with the accession number PRJCA027584.
